# Supplementary material for: Impact of Spirulina maxima Intake and Exercise (SIE) on Metabolic and Fitness Parameters in Sedentary Older Adults with Excessive Body Mass: Study Protocol of a Randomized Controlled Trial
Source: Int J Environ Res Public Health. 2021 Feb 8;18(4):1605. doi: 10.3390/ijerph18041605 (PMC7914563; doi:10.3390/ijerph18041605)
Supplement: Supplementary file 1 [file ijerph-18-01605-s001.zip › Supplementary Files/Supplementary File 2.pdf]

Supplementary File 2: Researchers ISAK certificates.

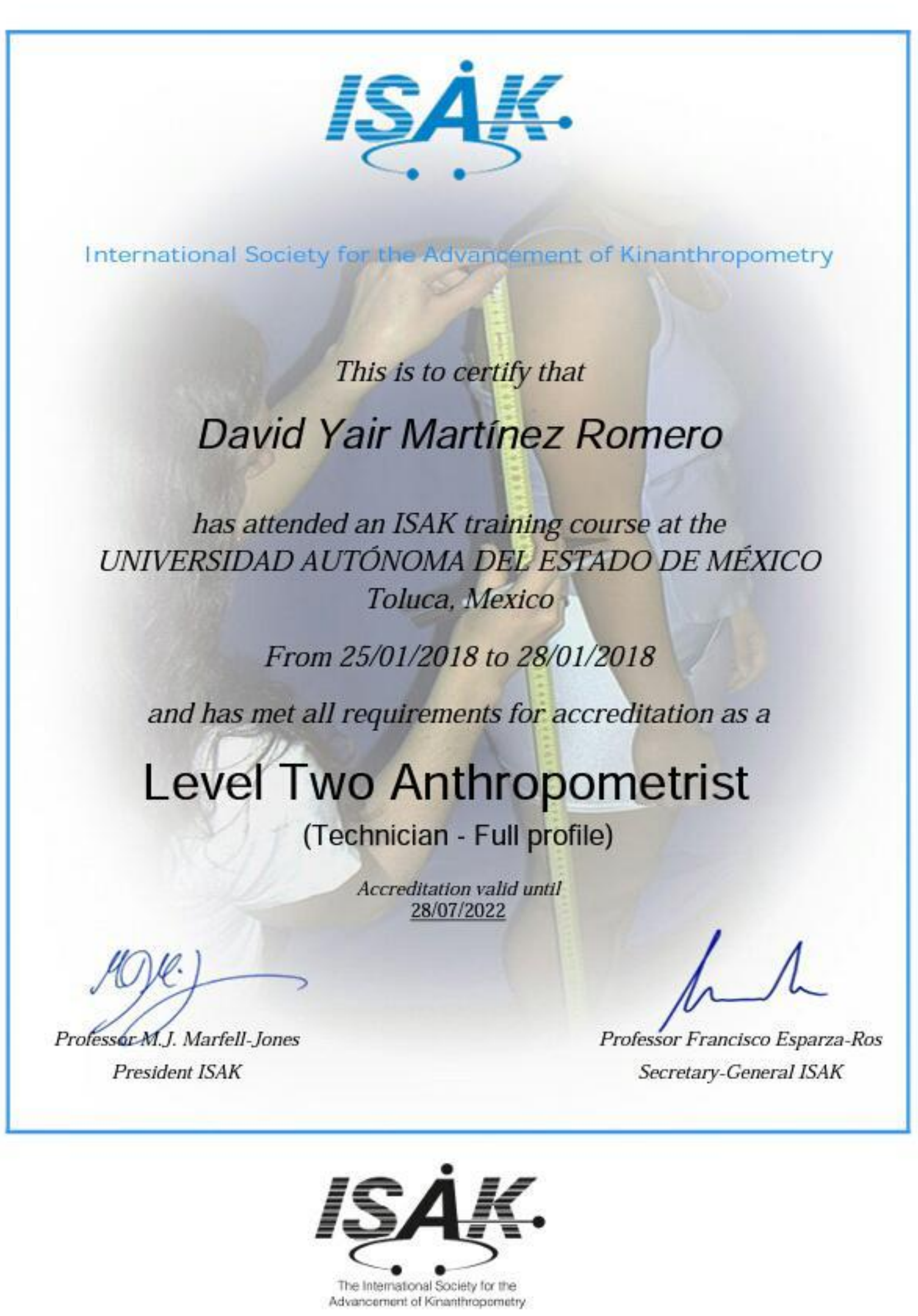

ISAK

International Society for the Advancement of Kinanthropometry

This is to certify that

David Yair Martínez Romero

has attended an ISAK training course at the
UNIVERSIDAD AUTÓNOMA DEL ESTADO DE MÉXICO
Toluca, Mexico

From 25/01/2018 to 28/01/2018

and has met all requirements for accreditation as a

Level Two Anthropometrist

(Technician - Full profile)

Accreditation valid until
28/07/2022

Professor M.J. Marfell-Jones
President ISAK

Professor Francisco Esparza-Ros
Secretary-General ISAK

ISAK

The International Society for the Advancement of Kinanthropometry

International Society for the Advancement of Kinanthropometry

This is to certify that

Marco Antonio Hernández Lepe

has attended an ISAK training course at the
Grupo Servicios de Salud Integral, Nutrición y Alimentación (Grupo SSINA), S.C.
Chihuahua, Mexico

From 19/01/2018 to 21/01/2018

and has met all requirements for accreditation as a

Level One Anthropometrist

(Technician - Restricted profile)

Accreditation valid until
21/05/2022

Professor M.J. Marfell-Jones	*Professor Francisco Esparza-Ros*
President ISAK	*Secretary-General ISAK*

The International Society for the Advancement of Kinanthropometry
