# Supplementary material for: Impact of Spirulina maxima Intake and Exercise (SIE) on Metabolic and Fitness Parameters in Sedentary Older Adults with Excessive Body Mass: Study Protocol of a Randomized Controlled Trial
Source: Int J Environ Res Public Health. 2021 Feb 8;18(4):1605. doi: 10.3390/ijerph18041605 (PMC7914563; doi:10.3390/ijerph18041605)
Supplement: Supplementary file 1 [file ijerph-18-01605-s001.zip › Supplementary Files/Supplementary File 4.pdf]

UNIVERSIDAD AUTÓNOMA DE BAJA CALIFORNIA

FACULTAD DE MEDICINA Y PSICOLOGÍA

Tijuana, B.C., a 30 de noviembre del 2020
Oficio, No. 998/2020-2

DR. MARCO ANTONIO HERNÁNDEZ LEPE
PRESENTE.-

Por medio de este conducto, me permito informarle que se sometió a revisión a la Comisión de Bioética de esta Facultad, la solicitud de revisión del proyecto de investigación titulado: **"EFFECT OF A SYSTEMATIC PHYSICAL EXERCISE PROGRAM AND SPIRULINA MAXIMA SUPPLEMENTATION ON THE BODY COMPOSITION, PHYSICAL FUNCTION AND BLOOD LIPID PROFILE IN SEDENTARY OLDER ADULTS WITH EXCESS WEIGHT: A DOUBLE-BLIND, RANDOMIZED, CROSSOVER, PLACEBO CONTROLLED TRIAL"**, el cual tuvo como resultado el siguiente dictamen: **APROBADO** (se anexa).

Sin más por el momento, me despido de usted enviándole un cordial saludo.

ATENTAMENTE
¨POR LA REALIZACIÓN PLENA DEL HOMBRE¨

DRA. JULIETA YADIRA ISLAS LIMÓN
DIRECTORA

UNIVERSIDAD AUTÓNOMA DE BAJA CALIFORNIA
FACULTAD DE MEDICINA Y PSICOLOGÍA
CAMPUS TIJUANA

C.c.p.- Dra. Ana Laura Martínez Martínez.- Coord. Inves y Posgrado FMP
C.c.p.- Dirección
